# Comparison of machine learning methods for prediction of venous thromboembolism among hospitalized adults

**DOI:** 10.1002/jhm.70342

**Published:** 2026-05-14

**Authors:** Yeji Ko, Benjamin French, Jonathan S. Schildcrout, Merna El‐Rifai, Adam Wright, Benjamin F. Tillman, Amanda S. Mixon, Henry J. Domenico

**Affiliations:** ^1^ Department of Biostatistics Vanderbilt University Medical Center Nashville Tennessee USA; ^2^ Department of Medicine Vanderbilt University Medical Center Nashville Tennessee USA; ^3^ Department of Biomedical Informatics Vanderbilt University Medical Center Nashville Tennessee USA; ^4^ Department of Medicine, Division of Hematology and Oncology Vanderbilt University Medical Center Nashville Tennessee USA; ^5^ Geriatric Research Education and Clinical Center, VA Tennessee Valley Healthcare System Nashville TN USA; ^6^ Department of Medicine, Division of General Internal Medicine and Public Health Vanderbilt University Medical Center Nashville Tennessee USA

## Abstract

**Background:**

Hospital‐acquired venous thromboembolism (HA‐VTE) is a significant cause of morbidity and mortality among hospitalized adults. Accurate prediction of HA‐VTE is crucial for timely intervention and prevention. While logistic regression is widely used for the development of clinical prediction models, there is ongoing interest in the potential for machine learning methods to enhance prediction performance.

**Objectives:**

This study aimed to identify the most practical method for the prediction of HA‐VTE based on electronic health record (EHR) data.

**Methods:**

We evaluated and compared the performance of prognostic models developed using logistic regression, random forest, extreme gradient boosting (XGBoost), deep neural networks, and an ensemble method to predict HA‐VTE using EHR data from a large academic medical center. Models were evaluated in a temporal external validation cohort based on discrimination and calibration metrics, overall and among key patient subgroups.

**Results:**

All models demonstrated similarly high discrimination, with *C* statistics ranging from 0.886 to 0.900. Logistic regression, random forest, and XGBoost showed excellent calibration, whereas deep neural networks exhibited poorer calibration. Additionally, prediction accuracy at a probability cut‐off of 0.02 and correlations between models indicated that logistic regression performed comparably well, if not better, compared with machine learning methods. Subgroup analyses further confirmed that the logistic regression model demonstrated strong discrimination and calibration among patient subpopulations.

**Conclusions:**

Our findings suggest that logistic regression is a highly effective tool for EHR‐based prediction of HA‐VTE.

## INTRODUCTION

Since enactment of the Health Information Technology for Economic and Clinical Health (HITECH) Act in 2009, which mandated that eligible healthcare providers adopt and demonstrate meaningful use of electronic health records (EHRs) by January 1, 2014, EHR data have been used to develop a multitude of risk‐prediction models.^1^ Concurrently, the development of methods for machine learning and artificial intelligence, along with exponential increases in computing power, have facilitated the development of complex risk‐prediction models using large EHR datasets.^2^ However, it has become generally accepted among methodologists that in settings with a finite number of established risk factors and well‐characterized clinical events, risk‐prediction models developed using conventional regression methods and machine learning methods generally exhibit similar prediction performance. Many recent comparative studies and systematic reviews have reported similar performance of logistic regression and machine learning models in settings with well‐defined clinical events and a moderate number of potential predictors.^3^, ^4^, ^5^, ^6^, ^7^, ^8^, ^9^, ^10^ As hospitals increasingly adopt tools based on complex machine learning methods in inpatient care, it remains unclear whether these methods meaningfully improve risk stratification for hospital‐acquired venous thromboembolism (HA‐VTE).

Venous thromboembolism (VTE)—a condition characterized by the formation of blood clots in the deep venous system as well as embolism to the pulmonary vasculature—has become an increasing concern in hospital settings.^11^, ^12^ Reducing risk of HA‐VTE, which is the leading cause of preventable in‐hospital death, is a major patient safety priority requiring timely preventative measures, such as pharmacologic or mechanical thromboprophylaxis.^13^ Identifying high‐risk patients for intervention, while excluding those at low risk, highlights the crucial need for accurate risk‐assessment tools.^14^ Despite conventional knowledge regarding the similar prediction performance of logistic regression and machine learning models, the current clinical landscape for prediction of HA‐VTE includes a multitude of prognostic models developed using many different, overly complex, statistical and machine learning methods.^15^, ^16^, ^17^, ^18^, ^19^, ^20^, ^21^ Overall, there is no single model that consistently outperforms the others for the prediction of HA‐VTE.^22^, ^23^ This fragmented landscape leads to vast uncertainty about which model to implement in routine clinical care, while rates of HA‐VTE continue to increase.

We previously developed and validated a prognostic model for HA‐VTE, dubbed VTE‐AI, among a large, heterogeneous population of hospitalized adults at an academic medical center using logistic regression with 25 predictors.^24^ This automated, real‐time prognostic model could identify HA‐VTE more rapidly within the EHR compared with widely used risk‐assessment tools such as Padua, Caprini, and international medical prevention registry on venous thromboembolism (IMPROVE) VTE scores.^25^, ^26^, ^27^ In addition, the model performed well overall and among subgroups of the inpatient population, in contrast to current models that are limited to specific patient subpopulations (e.g., patients undergoing surgery, patients with a history of cancer). Because timely identification of high‐risk patients is central to implementing thromboprophylaxis and preventing HA‐VTE, our current study sought to systematically compare different modeling approaches—logistic regression, random forests, extreme gradient boosting (XGBoost), and deep neural networks—to identify the most practical method for EHR‐based prediction of HA‐VTE.

## METHODS

### Data elements

Retrospective data on all individuals ≥16 years old admitted to Vanderbilt University Medical Center (VUMC) between January 1, 2018, and June 30, 2022, were obtained from VUMC's Research Derivative—a database of clinical data derived from the electronic data warehouse and restructured for research.^24^ For development of the prognostic models, we defined a derivation cohort of inpatient hospitalizations (or, “encounters”) between January 1, 2018, and December 31, 2020 (*n* = 132,330). Each encounter was treated as an independent event. For temporal external validation of the models, we defined a validation cohort of inpatient encounters between January 1, 2021, and June 30, 2022 (*n* = 62,546). The derivation and validation cohorts included 9259 overlapping patients. This study was approved by the Institutional Review Board of VUMC (#210161).

As detailed in our prior publication, events of HA‐VTE were identified using International Classification of Diseases, Tenth Revision codes during each eligible encounter.^24^ In the derivation cohort, there were 1187 HA‐VTEs (9.0 per 1000 encounters). A pool of 82 candidate predictors was selected based on real‐time availability in the EHR and prior known association with VTE, and included demographic and clinical characteristics, diagnostic procedures, vital signs, and laboratory measurements (Supporting Information S1: Table 1). We purposefully did not prescreen variables via automated selection procedures to allow the models to fully leverage all potential information in the data. To avoid the creation or exacerbation of disparities, we did not include race or ethnicity as candidate predictors. To avoid introducing confounding by indication, we did not include thromboprophylactic interventions as candidate predictors, as these likely reduce the risk of HA‐VTE among high‐risk patients, which would influence model performance. Consistent with our prior analysis, categorical risk factors with unknown values included “unknown” as a separate category; unknown values for continuous risk factors were imputed using the median value in the derivation cohort.^28^

### Sample size requirements

For the binary outcome of HA‐VTE with an approximate prevalence of 0.009, a hypothesized *C* statistic of 0.9, and 82 potential predictors (Supporting Information S1: Table 1), the minimum required sample size for developing a multivariable model using logistic regression is estimated at 26,523 encounters.^29^ With 132,330 encounters in the derivation cohort and 62,546 in the validation cohort, our study exceeds this requirement, which supports our ability to develop prognostic models with many variables even though the outcome is rare.

### Statistical analysis

#### Model development and optimization

We developed prognostic models for HA‐VTE in the derivation cohort using methods that represented a broad spectrum of commonly used regression and machine learning approaches^3^: logistic regression (regression‐based), random forest (tree‐based), XGBoost (gradient boosting), and deep neural networks. Each model was allowed to use the full set of 82 predictors. In addition to the individual models, we developed an ensemble model by averaging the predictions from the four individual models to estimate a final predicted probability. Full details on machine learning methods and software are provided in the supplement (Supporting Information S1: Table 2).

#### Model evaluation and performance comparison

We evaluated the prediction performance of the models in the validation cohort based on discrimination and calibration. Discrimination assessed a model's ability to distinguish between individuals with and without HA‐VTE and was quantified by the *C* statistic, which is equivalent to the area under the receiver operating characteristic curve for binary outcomes. A *C* statistic of 1 indicates perfect discrimination, whereas a *C* statistic of 0.5 indicates no discrimination.

Calibration assessed how well a model's predicted risks matched the risks in the observed data.^30^ It was evaluated visually using logistic calibration curves and quantified numerically using the calibration intercept, calibration slope, and the integrated calibration index (ICI). Both parametric and nonparametric calibration curves were displayed on the probability scale, as well as the log‐odds scale; plotting on the log‐odds scale reduced the impact of outlying extreme values. The parametric calibration intercept and calibration slope were estimated by fitting a logistic regression model of HA‐VTE outcomes against the predicted probabilities on the log‐odds scale.^31^, ^32^ The calibration intercept assessed overall over‐ or under‐estimation of risk; a calibration intercept < 0 indicates over‐estimation of risk (i.e., predicted probabilities are too large), whereas a calibration intercept > 0 indicates under‐estimation (i.e., predicted probabilities are too small). The calibration slope evaluated the extremity of predicted probabilities; a slope < 1 indicates that the predicted probabilities are too extreme (i.e., over‐estimating risk for high‐risk patients and under‐estimating it for low‐risk patients), while a slope > 1 indicates that predicted probabilities are too moderate (i.e., under‐estimating risk for high‐risk patients and over‐estimating risk for low‐risk patients). Supporting Information S1: Figure 1 provides a didactic illustration. The ICI represents the weighted difference between observed and predicted probabilities, in which observations are weighted based on the distribution of predicted probabilities; an ICI of 0 indicates perfect calibration. The Brier score, which calculates the mean squared difference between predicted probabilities and observed outcomes, was used as a combined measure of discrimination and calibration; a Brier score of 0 indicates perfect accuracy.

We assessed model accuracy at a cutoff of 0.02 for the predicted probability of HA‐VTE using sensitivity, specificity, positive predictive value (PPV), and negative predictive value (NPV). The 0.02 cutoff, although arbitrary, was determined to be a clinically meaningful threshold for risk of HA‐VTE, above which medical intervention should be considered. This threshold for intervention approaches a value that falls between the 3‐month VTE rates in the low‐ and high‐risk patients in the Padua model, and the risk level in the Caprini model at which pharmacologic prophylaxis is suggested, and the inflection point between intermediate‐to‐high in the IMPROVE VTE design.^21^, ^22^, ^23^ Most guidelines suggest that patients in this range with moderate‐to‐high risk should be evaluated for appropriateness of interventions, and that low‐risk patients should not have interventions.^33^ Note that the *C* statistic quantifies model discrimination across all possible dichotomizations for the predicted probabilities.

We graphically explored the distribution of predicted probabilities for each model and the correlation of predictions among the models. We examined variable importance for models developed using logistic regression, XGBoost, and random forest to identify the variables most useful for the prediction of HA‐VTE. The methods for determining variable importance varied by the R packages used. Deep neural networks did not provide variable importance metrics because they use a set of predictors rather than individual predictors in the model development. Finally, we conducted subgroup analyses based on admission type, age, sex, race, ethnicity, and history of metastatic cancer to assess model performance in potentially vulnerable patient groups. We adhered to transparent reporting of a multivariable prediction model for individual prognosis or diagnosis (TRIPOD) + AI guidelines.^34^

## RESULTS

Table 1 provides summary statistics for the patient characteristics used to identify subgroups in the derivation and validation cohorts; summaries for all potential predictors are provided in Supporting Information S1: Table 1. The demographic characteristics of patients in the derivation and validation cohorts were similar. The distribution of predicted probabilities obtained from each model, as well as their correlations across models, are provided in Supporting Information S1: Figure 2 and Figure 3, respectively. For all models, most predicted probabilities were less than 0.1, and except for the predicted probabilities estimated using random forest, were highly correlated across models (0.92–0.97).

**Table 1:** Cohort description.

	Derivation cohort (*N* = 132,330)		Validation cohort (*N* = 62,546)	
	Hospital‐acquired venous thromboembolism		Hospital‐acquired venous thromboembolism	
	Yes (*N* = 1187)	No (*N* = 131,143)	Yes (*N* = 864)	No (*N* = 61,682)
Age group				
Age <60 years	662 (55.8%)	76137 (58.1%)	474 (54.9%)	35705 (57.9%)
Age ≥60 years	525 (44.2%)	55006 (41.9%)	390 (45.1%)	25977 (42.1%)
Sex, no. (%)				
Female	457 (38.5%)	68,792 (52.5%)	361 (41.8%)	32,562 (52.8%)
Male	729 (61.4%)	62,349 (47.5%)	503 (58.2%)	29,113 (47.2%)
Unknown	1 (0.1%)	2 (<0.1%)	0 (0%)	7 (<0.1%)
Race, no. (%)				
Asian or Pacific Islander	11 (0.9%)	2319 (1.8%)	12 (1.4%)	960 (1.6%)
African American	238 (20.1%)	21,848 (16.7%)	174 (20.1%)	10,133 (16.4%)
American Indian or Alaskan Native	2 (0.2%)	328 (0.3%)	3 (0.3%)	189 (0.3%)
White	896 (75.5%)	101930 (77.7%)	643 (74.4%)	47347 (76.8%)
Unknown	40 (3.4%)	4718 (3.6%)	32 (3.7%)	3053 (4.9%)
Ethnicity, no. (%)				
Hispanic	39 (3.3%)	4993 (3.8%)	20 (2.3%)	2662 (4.3%)
Non‐hispanic	1123 (94.6%)	124,366 (94.8%)	819 (94.8%)	57,936 (93.9%)
Unknown	25 (2.1%)	1784 (1.4%)	25 (2.9%)	1084 (1.8%)
Admission type, no. (%)				
Elective	149 (12.6%)	31,401 (23.9%)	113 (13.1%)	14882 (24.1%)
Emergency	575 (48.4%)	66,837 (51.0%)	348 (40.3%)	28,248 (45.8%)
Trauma	82 (6.9%)	2394 (1.8%)	147 (17.0%)	4543 (7.4%)
Urgent	374 (31.5%)	29,988 (22.9%)	256 (29.6%)	13,942 (22.6%)
Unknown	7 (0.6%)	523 (0.4%)	0 (0%)	67 (0.1%)

### Discrimination in validation cohort

In the validation cohort, all models performed similarly well, with *C* statistics ranging from 0.886 to 0.900, indicating high discrimination accuracy (Table 2). The model developed using random forest achieved the lowest *C* statistic (0.886), while the model developed using the ensemble method achieved the highest *C* statistic (0.900). Despite some models (e.g., logistic regression and XGBoost) appearing to outperform others, their discrimination accuracy was qualitatively similar.

**Table 2:** Prediction performance metrics in the derivation and validation cohorts.

Model	Derivation cohort		Validation cohort				
	*C* statistic	Bootstrap *C* statistic^a^	*C* statistic	Integrated calibration index	Parametric calibration intercept^b^	Parametric calibration slope^b^	Brier score
Logistic regression	0.896	0.881	0.894	0.0037	0.201	0.983	0.0122
Random forest	0.993	0.884	0.886	0.0030	0.112	0.951	0.0124
XGBoost	0.908	0.889	0.893	0.0044	0.191	0.971	0.0122
Neural network	0.909	‐	0.890	0.0054	0.162	0.880	0.0123
Ensemble method	0.975	‐	0.900	0.0043	0.466	1.038	0.0121

^a^ A bootstrap‐based approach on the derivation cohort data was used to control overfitting; this approach was not available for the models developed using neural networks and the ensemble method.

^b^ Calibration intercept was calculated as the log‐odds of the outcome when the predicted probability is 0.5 (i.e., the predicted log odds is 0) and the calibration slope as the change in the log‐odds of the outcome for a one‐unit increase in the predicted probability. The calibration intercept assessed overall over‐ or under‐estimation of risk; a calibration intercept < 0 indicates over‐estimation of risk (i.e., predicted probabilities are too large), whereas a calibration intercept > 0 indicates under‐estimation (i.e., predicted probabilities are too small). The calibration slope evaluated the extremity of predicted probabilities; a slope < 1 indicates that the predicted probabilities are too extreme (i.e., over‐estimating risk for high‐risk patients and under‐estimating it for low‐risk patients), while a slope > 1 indicates that predicted probabilities are too moderate (i.e., under‐estimating risk for high‐risk patients and over‐estimating risk for low‐risk patients); see Supporting Information S1: Figure 1 for an illustration.

### Calibration in validation cohort

Figure 1 demonstrates overall good calibration for most models, except for the models developed using deep neural networks and the ensemble method. For the models developed using logistic regression, random forest, and XGBoost, the observed probabilities were approximately 0.5 when the predicted probability was 0.5 (Figure 1a). In addition, their calibration curves were closely aligned with the 45‐degree line (Figure 1b). Both the logistic regression and XGBoost models had calibration intercepts around 0.2 and calibration slopes close to 1 (Table 2). Although the random forest model had a calibration slope further away from 1, its calibration intercept (0.112) was much lower than that of the logistic regression and XGBoost models. In contrast, the model developed using deep neural networks was poorly calibrated, as evidenced by a calibration slope well below 1, meaning that the model overestimated risk for high‐risk patients and underestimated it for low‐risk patients.

**Figure 1:**
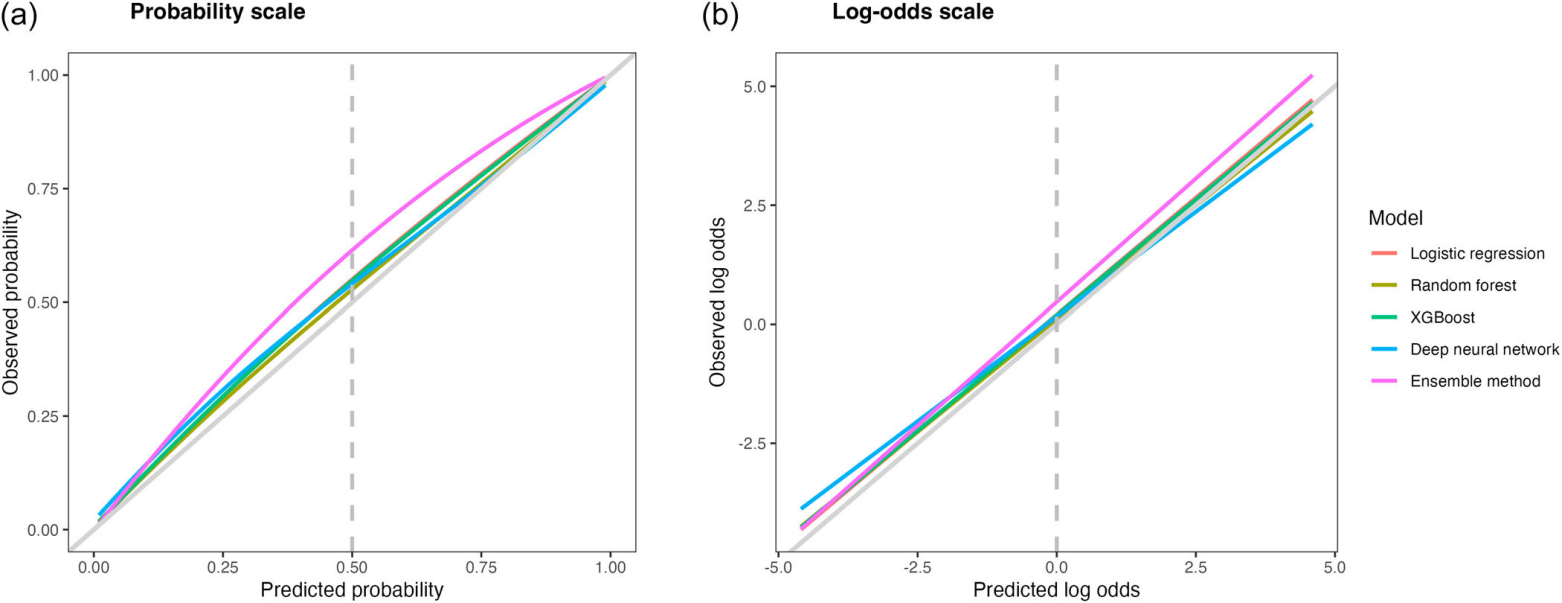
Calibration curves on the (a) probability scale and (b) log‐odds scale. The gray vertical dashed line corresponds to the point at which the calibration intercept is calculated. The gray 45‐degree line corresponds to a calibration intercept of 0 and a calibration slope of 1 (i.e., perfect calibration).

The ensemble model performed differently compared with the individual models. Unlike the individual models, the ensemble model had a calibration slope greater than 1, meaning that the model under‐estimated risk for high‐risk patients and over‐estimated risk for low‐risk patients. This difference could be due to the averaging effect of combining higher predictions above 0.5 with lower predictions from other models, such as a random forest. The ensemble model also had a high calibration intercept (0.466), meaning that the model generally under‐estimated risk. Nonparametric calibration curves provided in Supporting Information S1: Figure 4 showed that the logistic regression model had the best calibration overall. Regarding the Brier score, no substantial differences were observed between the five models.

### Model performance at 0.02 cutoff

The random forest model predicted the highest number of patients with a probability of HA‐VTE greater than 0.02 (*n* = 8696; 13.9%), while the neural network model identified the lowest number of patients (*n* = 5465; 8.7%) (Table 3). At the 0.02 cutoff, all models showed sensitivities around 0.7, correctly classifying roughly 70% of patients with HA‐VTE as cases. The random forest and ensemble models had slightly higher values for sensitivity (0.710 and 0.707, respectively) compared with others, which were less than 0.7. PPVs were comparable across the models, with most around 0.1, indicating that approximately 10% of patients with a predicted probability greater than 0.02 developed HA‐VTE. The random forest model had the lowest PPV (0.077). NPVs were close to 1 for all models, suggesting that almost all patients classified as control did not have HA‐VTE.

**Table 3:** Model accuracy measures at a probability cut‐off of 0.02.

Model	Patients with predicted HA‐VTE probability > 0.02 *N* (%)	Sensitivity^a^	Specificity^b^	PPV^c^	NPV^d^
Logistic regression	6508 (10.4%)	0.690	0.904	0.092	0.995
Random forest	8696 (13.9%)	0.710	0.880	0.077	0.995
XGBoost	5911 (9.5%)	0.670	0.914	0.098	0.995
Neural network	5465 (8.7%)	0.640	0.920	0.101	0.995
Ensemble method	6599 (10.6%)	0.707	0.903	0.093	0.996

^a^ Sensitivity is the proportion of patients who were classified as a case (i.e., predicted probability > 0.02) among patients who developed hospital‐acquired venous thromboembolism (HA‐VTE).

^b^ Specificity is the proportion of patients who were classified as a control (i.e., predicted probability < 0.02) among patients who did not develop HA‐VTE.

^c^ Positive predictive value (PPV) is the proportion of patients who developed HA‐VTE among predicted cases (i.e., predicted probability > 0.02).

^d^ Negative predictive value (NPV) is the proportion of patients who did not develop HA‐VTE among predicted controls (i.e., predicted probability < 0.02).

### Variable importance in different models

The 10 most‐important predictors in the logistic regression and XGBoost models were similar (Table 4). In fact, the five most important variables—presence of a central line, cardiac arrhythmia, fluid electrolyte disorder, hypoxemia, and pneumonia—are identical in both models. On the contrary, the random forest model identified different important variables, potentially due to its distinct methods used for evaluating variable importance. For instance, while the central line is the most important variable in both the logistic regression and XGBoost models, it was not among the top 10 predictors in the random forest model. Oxygen saturation, temperature, and chloride level are important in the random forest model but are not considered as important in the logistic regression or XGBoost models.

**Table 4:** Top‐10 most important variables.

Rank	Logistic regression	Random forest	XGBoost
1	Central line	Pneumonia	Central line
2	Cardiac arrhythmia	C‐reactive protein	Cardiac arrhythmia
3	Fluid electrolyte disorder	Hypoxemia	Fluid electrolyte disorder
4	Hypoxemia	Weight loss	Hypoxemia
5	Pneumonia	Oxygen saturation	Pneumonia
6	Weight loss	Pleural disease	Coagulopathy
7	Coagulopathy	Coagulopathy	Weight loss
8	C‐reactive protein	Temperature	C‐reactive protein
9	Pleural disease	Chloride	Pleural disease
10	Acute renal failure	Cardiac arrhythmia	White blood cell count

### Subgroup analyses

Among five different models, the ensemble model consistently had the highest *C* statistic across all subgroups, while the random forest model consistently had the lowest *C* statistic (Supporting Information S1: Figure 5). The logistic regression, XGBoost, and deep neural network models performed similarly well in terms of discrimination, yet logistic regression performed slightly better among some subgroups, such as Black or Hispanic patients. The logistic regression and XGBoost models tended to have calibration slopes closest to 1 across subgroups (Supporting Information S1: Figure 6). In contrast, the deep neural network model showed poor subgroup calibration, with slopes well below 1. Interestingly, the Asian or Pacific Islander group has the highest *C* statistic, but its calibration slope is farthest from 1, possibly due to the small sample size.

## DISCUSSION

We systematically compared different modeling approaches—logistic regression, random forests, extreme gradient boosting, and deep neural networks—to identify the most practical method for EHR‐based prediction of HA‐VTE. With respect to multiple measures of discrimination and calibration, logistic regression performed as well as, if not better than, machine learning methods. Our approach was purposefully designed to allow machine learning methods the opportunity to perform well. In particular, a purported strength of machine learning methods is their ability to uncover hidden patterns through high‐dimensional, nonlinear interactions, unlike logistic regression, for which interactions and nonlinear associations must be explicitly modeled.^2^ We conducted our study among a large, heterogeneous patient population, and included many potential predictors to enable identification of such patterns. Despite this, machine learning methods did not perform better than logistic regression, neither overall nor among subgroups. Our results are similar to a prior study with a much smaller sample size.^35^ In future work, we will conduct a pragmatic randomized trial to evaluate whether use of clinical decision support tools based on such a logistic regression model for HA‐VTE improves patient outcomes.

The current landscape for model‐based prognosis of HA‐VTE is highly fragmented.^22^, ^23^ Yet models developed for specific patient subpopulations with different statistical and machine learning methods continue to appear in the literature: an XGBoost model for patients with cancer, a random forest model for patients in the intensive care unit, and an XGBoost model for orthopedic patients, among others.^15^, ^16^, ^17^, ^18^, ^19^, ^20^, ^21^ This fragmented landscape leads to vast uncertainty regarding which model to implement for the prediction and prevention of HA‐VTE. In the face of such uncertainty, institutions fall back on potentially outdated and inaccurate prognostic models. For example, the Padua score, which is implemented at our institution, has been shown to have poor prognostic performance, underperforms with attempts at EHR integration and automation, and fails to reduce VTE or mortality when evaluation of the score is obligatory, even when prophylaxis rates increase.^36^, ^37^, ^38^ Our prior and current work has shown that a conventional logistic regression model can perform just as well as, if not better than, machine learning models for the prediction of HA‐VTE, both overall and among key subgroups of the adult inpatient population.

For automated EHR‐based implementation of prognostic models in daily clinical practice, logistic regression offers simplicity, interpretability, and ease of integration. For example, in VUMC's Epic, a logistic regression can be implemented directly in the EHR as a formula‐based scoring system, allowing it to process data in real time with minimal computational overhead and no custom programming required. Epic also allows for some, but not all, random forests to be run directly in the EHR. The other algorithms we evaluated—XGBoost and deep neural networks—can also be implemented in Epic, but require the use of an external, cloud‐based system called Nebula. Once implemented, how the model's output is best communicated to providers is an open question.^39^ We are currently conducting observations and interviews with providers to determine their preferences for how patients' risk is assessed and acted upon.

Clinical judgment and provider autonomy must be respected when implementing a prognostic model. A prognostic model merely integrated into the EHR is not guaranteed to prevent HA‐VTE, as shown by implementation of the Padua score.^36^, ^37^, ^38^ Beyond adequate prediction performance, the model's content must have face validity for clinicians to trust and utilize it in their daily work.^40^, ^41^ From the top 10 most important variables we identified, a clinician might question why chloride in the random forest model would be predictive of HA‐VTE. In looking at the top five most important variables, all are indicative of more severe illness in general. Our interviews with providers have, therefore, provided additional clinically relevant variables (e.g., history of cancer) that will be included in the model to be tested in a randomized trial. Even with a model acceptable to and understood by clinicians, clinicians will be presented with a predicted probability, at which point they can still exercise their clinical judgment regarding the need for prophylaxis. In our randomized trial, we will not remove the decision to order prophylaxis or not from the clinicians. We believe clinicians have a more holistic view of the patients they are treating than any mathematical model. A prognostic model is a tool to aid in decision‐making, hopefully making it easier to decipher which patients are lower versus higher risk and perhaps reduce over‐ or under‐prophylaxis in the hospitalized population. Successful integration of the model into the EHR will require engagement of hospital teams, iterative refinement of the model's display, and minimization of alert fatigue while continuing to safely surveil.

We postulate that automated implementation of a dynamic prognostic model in the EHR can improve rates of appropriate thromboprophylaxis and reduce the incidence of HA‐VTE. Our institution's implementation of the Padua model requires the provider to manually calculate the risk score and then decide about prophylaxis in an order set. An automated risk calculation, for which variables are automatically pulled from the EHR, will streamline the workflow and remove the provider burden of manual scoring requirements. Prior literature has demonstrated that active clinical decision support that presents calculated risk or interruptive alerts tends to be more effective at engaging clinicians than passive alerts alone.^42^ Automated and individualized risk prediction also has the potential to reduce the racial and sex disparities in prophylaxis provision while improving rates of appropriate prophylaxis.^40^ When a risk score is embedded into the EHR and tooled as a required step in patient admission, it increases adoption.^43^ Our model has the added strength of dynamic updates to reflect the most up‐to‐date HA‐VTE risk for each patient, which may lead to changes in prophylaxis beyond admission. All these strengths combined are a substantial improvement over the current clinical workflow and prediction tools.^44^

We acknowledge the following limitations. The overall risk of HA‐VTE was very low in both the derivation and validation cohorts, meaning that patients without HA‐VTE vastly outnumbered HA‐VTE cases. This phenomenon, known as class imbalance, can be problematic when performing classification because models typically default to predicting the majority class, which is clinically less important.^45^ We addressed class imbalance in several ways. First, we used machine learning methods that put more emphasis on the minority class than standard logistic regression. Second, we focused on generating predicted probabilities, rather than predicting class membership. Third, for model evaluation, we used the *C* statistic rather than accuracy, as accuracy places more weight on the common classes than on rare classes.^40^ Of note, despite the class imbalance, the metrics for discrimination and calibration showed that most models provided acceptable predictions in the validation cohort, albeit with lower sensitivity at the 0.02 cutoff for the predicted probability of HA‐VTE.

Subgroup analysis revealed that models sometimes performed worse among higher risk subgroups. For example, all models exhibited lower discrimination ability among patients aged over 60 years, who are generally considered higher risk. This result could arise if our analysis did not include variables that were particularly important for these subgroups. Alternatively, this result suggests the need for further model refinement to improve prediction accuracy among high‐risk subgroups, although our prior analyses found no benefit from adding interactions with age or a history of cancer to logistic regression models.^24^ We are currently working to refine our VTE‐AI model by considering additional risk factors, including active medications, measures of mobility, and socio‐environmental factors, and to perform additional external validation, including geographic and domain validation.^46^

## CONCLUSION

In summary, we used EHR data on a broad population of hospitalized adults to develop, validate, and compare prognostic models for HA‐VTE using logistic regression and machine learning techniques. Based on our results, we are confident that logistic regression can provide reliable estimates for the probability of HA‐VTE. Implementation of an automated model in the EHR will simply the clinical workflow and guide clinical decision‐making for the appropriate initiation of thromboprophylaxis, with the potential for reduced rates of HA‐VTE.

## REPORTING CHECKLIST

We adhered to TRIPOD + AI guidelines.

## CONFLICT OF INTEREST STATEMENT

The authors declare no conflicts of interest.

## ETHICS STATEMENT

This study was approved by the Institutional Review Board of VUMC (#210161).

## Supporting information

VTE_ML_supplement.

## Data Availability

The data and materials that support the findings of this study are not openly available due to reasons of sensitivity and are available from the corresponding author upon reasonable request.
