# Supplementary material for: Comparison of machine learning methods for prediction of venous thromboembolism among hospitalized adults
Source: J Hosp Med. 2026 May 14;21(11):1218–26. doi: 10.1002/jhm.70342 (PMC13728529; doi:10.1002/jhm.70342)

Supplement to:

Comparison of Machine Learning Methods for Prediction of Venous Thromboembolism among Hospitalized Adults

Yeji Ko, Benjamin French, Jonathan S Schildcrout, Merna El-Rifai, Adam Wright, Benjamin F Tillman, Amanda S Mixon, Henry J Domenico

Supplementary Methods
Supplementary Table 1. Candidate predictors summarized for the derivation and validation cohorts
Supplementary Table 2. R packages, commands, and arguments for fitting logistic regression and machine learning models
Supplementary Figure 1. Illustration of calibration intercept and calibration slope
Supplementary Figure 2. Summary of predicted probabilities by model
Supplementary Figure 3. Correlation in predicted probabilities between models
Supplementary Figure 4. Non-parametric calibration curves on the (a) probability scale and (b) log-odds scale
Supplementary Figure 5. *C* statistics among key subgroups
Supplementary Figure 6. Calibration slopes among key subgroups

Supplementary Methods

Machine Learning Methods

We developed prognostic models for HA-VTE in the derivation cohort using logistic regression and various machine learning methods: random forest, extreme gradient boosting (XGBoost), and deep neural networks. Each model was allowed to use the full set of 82 predictors. Random forest and XGBoost are ensemble learning methods that potentially improve prediction performance and prevent overfitting by combining multiple decision trees [1,2]. Both random forest and XGBoost use random subsets of the dataset to construct each tree. With random forests, predictions are made by aggregating the results from all the trees, using majority voting for classification or averaging for regression [3]. On the other hand, XGBoost builds trees sequentially, minimizing the residual errors from previously trained trees. While random forests can be slow to train, especially with many trees and datasets, XGBoost is more efficient for large-scale data due to its parallelization capabilities even though tuning its hyperparameters can be more challenging. Deep neural networks, which emulate the complex functions of the human brain, consist of non-linear layers with processing units called neurons [4]. These networks can model complex relationships between input predictors and outputs. Despite their predictive power and efficiency, deep neural networks are often criticized for their limited interpretability [5].

The ‘caret’ package in R was used to develop models using logistic regression and XGBoost, while the R packages ‘randomForestSRC’ and ‘keras’ were used to develop models using random forest and deep neural networks, respectively (Supplementary Table 2). In the ‘randomForestSRC’ package, a quantile classifier was applied to optimize both true- positive and true-negative rates in the presence of class imbalance (i.e., many fewer cases of HA-VTE compared to non-cases) [6,7]. For random forest and XGBoost, optimal hyperparameters were selected based on *C* statistics with bootstrap and cross-validation. In contrast, for deep neural networks, hyperparameters such as the number of layers and neurons per layer were selected based on the number of predictors and sample size. Other hyperparameters in the deep neural networks were tuned manually through a range of possible hyperparameter values. Activation functions and loss functions were determined by the type of outcome in the model.

In addition to the individual models, we developed an ensemble model to potentially further enhance prediction performance. Ensemble learning combines multiple heterogeneous models, improving overall model performance [8,9]. This approach can be implemented through bagging, boosting, and stacking [10]. For our analysis, we used bagging, which involves averaging the predictions from the four individual models to estimate a final predicted probability.

[1] Breiman L. Random forests. Machine Learning 2001; 45(1):5-32.

[2] Chen T, Guestrin C. XGBoost: A scalable tree boosting system. Proceedings of the 22nd ACM SIGKDD International Conference on Knowledge Discovery and Data Mining 2016; 785-94.

[3] Scornet E. Random forests and kernel methods. IEEE Transactions on Information Theory 2016; 62(3):1485-500.

[4] Esteva A, Robicquet A, Ramsundar B, Kuleshov V, DePristo M, Chou K, Cui C, Corrado G, Thrun S, Dean J. A guide to deep learning in healthcare. Nature Medicine 2019; 25(1):24-9.

[5] Sargent DJ. Comparison of artificial neural networks with other statistical approaches: Results from medical data sets. Cancer 2001; 91(S8):1636-42.

[6] Kuhn M. Building predictive models in R using the caret package. Journal of Statistical Software 2008; 28(5):1-26.

[7] Ishwaran H, Kogalur UB, Kogalur MU. Package ‘randomForestSRC’. Breast 2022; 6(1).

[8] Polikar R. Ensemble learning. In: Ensemble Machine Learning: Methods and Applications. 2012;1-34.

[9] Hou S, Liu Y, Yang Q. Real-time prediction of rock mass classification based on TBM operation big data and stacking technique of ensemble learning. Journal of Rock Mechanics and Geotechnical Engineering 2022; 14(1):123-43.

[10] Sun W, Bayes T. A stacking ensemble learning framework for annual river ice breakup dates. Journal of Hydrology 2018; 561:636-50.

Supplementary Table 1. Candidate predictors summarized for the derivation and validation cohorts

	Derivation (N=132330)	Validation (N=62546)
**Age, years**		
Mean (SD)	53.6 (18.7)	53.5 (19.0)
Median [IQR]	55.8 [37.0, 68.4]	55.5 [36.5, 68.8]
**Age, group**		
Age < 60 years	76799 (58.0%)	36179 (57.8%)
Age ≥ 60 years	55531 (42.0%)	26367 (42.2%)
**Sex, No. (%)**		
Female	69249 (52.3%)	32923 (52.6%)
Male	63078 (47.7%)	29616 (47.4%)
Unknown	3 (<0.1%)	7 (<0.1%)
**Race[1], No. (%)**		
Asian or Pacific Islander	2330 (1.8%)	972 (1.6%)
African American	22086 (16.7%)	10307 (16.5%)
American Indian or Alaskan Native	330 (0.2%)	192 (0.3%)
White	102826 (77.7%)	47990 (76.7%)
Unknown	4758 (3.6%)	3085 (4.9%)
**Ethnicity[1], No. (%)**		
Hispanic	5032 (3.8%)	2682 (4.3%)
Non-Hispanic	125489 (94.8%)	58755 (93.9%)
Unknown	1809 (1.4%)	1109 (1.8%)
**Admission Type, No. (%)**		
Elective	31550 (23.8%)	14995 (24.0%)
Emergency	67412 (50.9%)	28596 (45.7%)
Trauma	2476 (1.9%)	4690 (7.5%)
Urgent	30362 (22.9%)	14198 (22.7%)

Unknown	530 (0.4%)	67 (0.1%)
**Body Mass Index, kg/m^2**		
Mean (SD)	29.4 (8.2)	29.6 (8.1)
Median [IQR]	28.1 [23.9, 33.3]	28.2 [24.0, 33.5]
Missing	9160 (6.9%)	4655 (7.4%)
**Temperature, °C**		
Mean (SD)	36.7 (0.6)	36.74 (0.6)
Median [IQR]	36.7 [36.5, 36.9]	36.7 [36.5, 36.9]
Missing	305 (0.2%)	457 (0.7%)
**Heart Rate, bpm**		
Mean (SD)	87.7 (21.7)	88.14 (21.9)
Median [IQR]	85 [72, 101]	86 [72, 101]
Missing	64155 (48.5%)	29677 (47.4%)
**Systolic Blood Pressure, mmHg**		
Mean (SD)	128.0 (23.1)	128.3 (22.7)
Median [IQR]	126 [112, 142]	126 [112, 142]
Missing	218 (0.2%)	408 (0.7%)
**White Blood Cell Count, 10^3/ mcL**		
Mean (SD)	10.8 (11.0)	10.8 (9.8)
Median [IQR]	9.5 [6.9, 12.9]	9.5 [6.9, 12.9]
Missing	13045 (9.9%)	8821 (14.1%)
**Red Blood Cell Count, 10^6/ mcL**		
Mean (SD)	4.1 (0.8)	4.0 (0.8)
Median [IQR]	4.1 [3.5, 4.6]	4.1 [3.5, 4.6]
Missing	13045 (9.9%)	8826 (14.1%)
**Red Cell Distribution Width, %**		
Mean (SD)	14.6 (2.4)	14.6 (2.5)

Median [IQR]	13.9 [13.0, 15.6]	13.9 [13.0, 15.6]
Missing	13174 (10.0%)	8879 (14.2%)
**Oxygen Saturation, %**		
Mean (SD)	72.2 (20.3)	70.8 (21.2)
Median [IQR]	75 [59, 89]	74 [56, 89]
Missing	113995 (86.1%)	51952 (83.1%)
**Sodium, mEq/L**		
Mean (SD)	137.4 (4.2)	137.1 (4.3)
Median [IQR]	138 [135, 140]	138 [135, 140]
Missing	21402 (16.2%)	13157 (21.0%)
**Mean Platelet Volume, fL**		
Mean (SD)	10.4 (1.1)	10.4 (1.1)
Median [IQR]	10.3 [9.6, 11.0]	10.3 [9.7, 11.1]
Missing	15355 (11.6%)	9984 (16.0%)
**Mean Cell Volume, fL**		
Mean (SD)	89.4 (7.1)	89.6 (7.2)
Median [IQR]	90 [86, 94]	90 [86, 94]
Missing	13050 (9.9%)	8826 (14.1%)
**Mean Cell Hemoglobin Concentration, mg/dL**		
Mean (SD)	32.9 (1.6)	33 (1.6)
Median [IQR]	33 [32, 34]	33 [32, 34]
Missing	13086 (9.9%)	8839 (14.1%)
**Potassium, mmol/L**		
Mean (SD)	4.2 (0.7)	4.3 (0.8)
Median [IQR]	4.1 [3.7, 4.5]	4.2 [3.8, 4.6]
Missing	20550 (15.5%)	13025 (20.8%)
**Chloride, mEq/L**		

Mean (SD)	104.2 (5.5)	103.8 (5.5)
Median [IQR]	105 [101, 108]	104 [101, 107]
Missing	22382 (16.9%)	13379 (21.4%)
**C-Reactive Protein, mg/dL**		
Mean (SD)	76.0 (88.0)	82.5 (89.2)
Median [IQR]	39.0 [10.2, 115.9]	50.6 [13.5, 124.4]
Missing	113367 (85.7%)	52957 (84.7%)
**Blood Urea Nitrogen, mg/dL**		
Mean (SD)	21.4 (18.5)	21.7 (18.8)
Median [IQR]	16 [11, 24]	16 [11, 25]
Missing	22389 (16.9%)	13388 (21.4%)
**Comorbidities, No. (%)**		
History of thrombosis[2]	7672 (5.8%)	3679 (5.9%)
Hypoxemia[2]	12825 (9.7%)	6631 (10.6%)
Central line[2]	3892 (2.9%)	2120 (3.4%)
Cardiac arrhythmia	49872 (37.7%)	24595 (39.3%)
Fluid electrolyte disorder	48235 (36.5%)	24422 (39.0%)
Dyslipidemia	39669 (30.0%)	21220 (33.9%)
Gastroesophageal reflux disease	37125 (28.1%)	18360 (29.4%)
Tobacco use	25010 (18.9%)	12197 (19.5%)
Complicated hypertension	30005 (22.7%)	14970 (23.9%)
Acute renal failure	27219 (20.6%)	13659 (21.8%)
Chronic pulmonary disease	24838 (18.8%)	12167 (19.5%)
Anxiety	22548 (17.0%)	13384 (21.4%)
Depression	21655 (16.4%)	8245 (13.2%)
Diabetes with chronic complications	23552 (17.8%)	12005 (19.2%)
Breathing symptoms	22602 (17.1%)	10340 (16.5%)

Other anemia	21585 (16.3%)	11550 (18.5%)
Congestive heart failure	23173 (17.5%)	11700 (18.7%)
Solid tumor without metastasis	20258 (15.3%)	9462 (15.1%)
Renal failure	24553 (18.6%)	12347 (19.7%)
Diabetes without chronic complications	19718 (14.9%)	8313 (13.3%)
Other psychiatric disorders	13448 (10.2%)	7563 (12.1%)
Myocardial infarction	15990 (12.1%)	7884 (12.6%)
Hypothyroidism	16727 (12.6%)	8430 (13.5%)
Cerebrovascular disease	12081 (9.1%)	5901 (9.4%)
Personal cancer history	15671 (11.8%)	7850 (12.6%)
Coagulopathy	14088 (10.6%)	7890 (12.6%)
Sleep apnea	15930 (12.0%)	8006 (12.8%)
Liver disease	13497 (10.2%)	6911 (11.0%)
Drug abuse	8944 (6.8%)	4098 (6.6%)
Pneumonia	10979 (8.3%)	5580 (8.9%)
Other neurological disorders	10027 (7.6%)	4611 (7.4%)
Arthritis	11016 (8.3%)	5193 (8.3%)
Metastatic cancer	9435 (7.1%)	4398 (7.0%)
Valvular disease	9968 (7.5%)	5369 (8.6%)
Alcohol abuse	6546 (4.9%)	3127 (5.0%)
Weight loss	8753 (6.6%)	3949 (6.3%)
Peripheral vascular disease	7748 (5.9%)	4126 (6.6%)
Epilepsy	7040 (5.3%)	3063 (4.9%)
Deficiency anemia	7177 (5.4%)	4785 (7.7%)
Pleural disease	6110 (4.6%)	2965 (4.7%)
Benign prostatic hypertrophy	5842 (4.4%)	3258 (5.2%)
Family history cancer	5693 (4.3%)	2675 (4.3%)

Abnormal lung imaging	4980 (3.8%)	2731 (4.4%)
Paralysis	4466 (3.4%)	2267 (3.6%)
Rheumatoid arthritis	5467 (4.1%)	2672 (4.3%)
Gout	5455 (4.1%)	2768 (4.4%)
Dementia	4064 (3.1%)	1823 (2.9%)
Psychoses	1291 (1.0%)	677 (1.1%)
Cough	2958 (2.2%)	470 (0.8%)
Osteoporosis	3223 (2.4%)	1660 (2.7%)
Lymphoma	2095 (1.6%)	938 (1.5%)
Peptic ulcer disease	2110 (1.6%)	948 (1.5%)
Blood loss anemia	1826 (1.4%)	966 (1.5%)
Cachexia	1643 (1.2%)	924 (1.5%)
Glaucoma	1826 (1.4%)	885 (1.4%)
Candidal stomatitis	1347 (1.0%)	571 (0.9%)
Intellectual disability	535 (0.4%)	260 (0.4%)
Sclerosis	738 (0.6%)	389 (0.6%)
Influenza	710 (0.5%)	124 (0.2%)
Venous disease	569 (0.4%)	320 (0.5%)
Macular degeneration	607 (0.5%)	257 (0.4%)
Secondary polycythemia	400 (0.3%)	255 (0.4%)
Cataracts	411 (0.3%)	280 (0.4%)
**Hospital-acquired Venous Thromboembolism, No. (%)**	1187 (0.9%)	864 (1.4%)

IQR, inter-quartile range; SD, standard deviation
[1] Neither race nor ethnicity were considered for inclusion in the prognostic model.
[2] Not included in the Elixhauser comorbidity index.

Supplementary Table 2. R packages, commands, and arguments for fitting logistic regression and machine learning models

Model	R package	Command	Requires dummy coding	Hyperparameters (argument)	Selected hyperparameter values
Logistic regression	*caret*	*glm*	No	• None	• None
Random forest	*randomforestSRC*	*rfsrc*	No	• Number of trees (ntree)<br>• Number of splits (mtry)<br>• Minimum number of observations in terminal nodes (nodesize)	• ntree = 3000<br>• mtry = 82<br>• nodesize = 30
XGBoost	*caret*	*xgbTree*	No	• Learning rate (eta)<br>• Maximum depth of tree (max_depth)<br>• Minimum split loss (gamma)<br>• Percentage of features for tree (colsample_bytree)<br>• Minimum sum of instance weight (min_child_weight)	• eta = 0.3<br>• max_depth = 2<br>• gamma = 0<br>• colsample_bytree = 0.8<br>• min_child_weight = 1
Neural network	*keras*	*keras_model_sequential*	Yes	• Number of layers (layer_dense)<br>• Number of neurons (units)<br>• Drop-out rates (layer_dropout)<br>• Activation function (activation)<br>• Loss function (loss), Optimizer (optimizer)<br>• Number of epochs (epochs), Batch size (batch_size)	• layer_dense = 4<br>• units = 85 -> 64 -> 32 -> 8<br>• layer_dropout = 0.2-0.4<br>• activation = relu and sigmoid<br>• loss = binary_crossentropy<br>• optimizer = adam<br>• epochs = 8<br>• batch_ size = 100

C statistic is optimized via bootstrap in *caret* and cross validation in *randomforestSRC* and *keras.*

Supplementary Figure 1. Illustration of calibration intercept and calibration slope

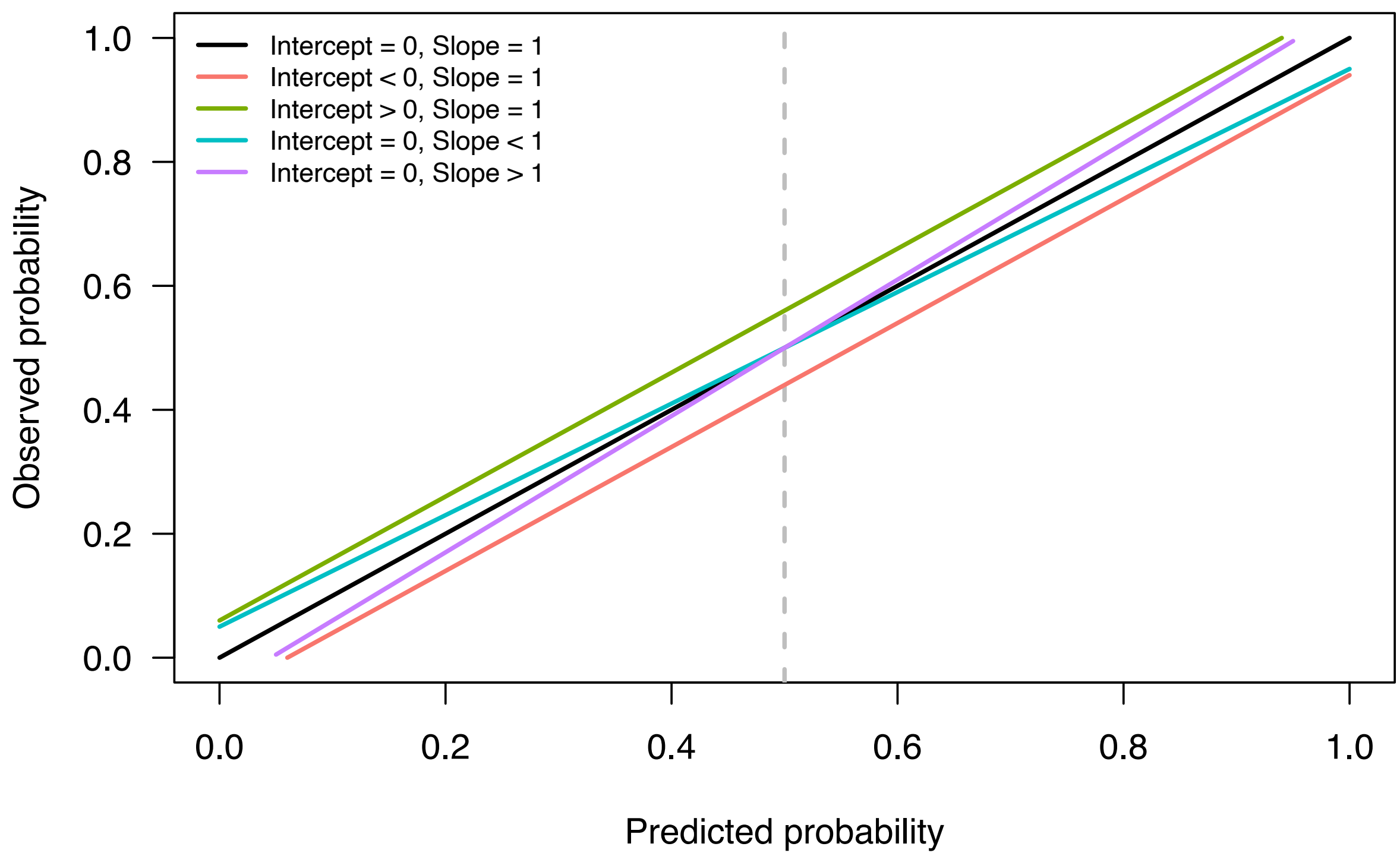

In the figure above:

- The gray vertical dashed line corresponds to the point at which the calibration intercept is calculated.
- The black line (intercept = 0, slope = 1) corresponds to perfect calibration.
- The red line (intercept < 0, slope = 1) corresponds to overall over-estimation of risk (that is, predicted probabilities are too large).
- The green line (intercept > 0, slope = 1) corresponds to overall under-estimation of risk (that is, predicted probabilities are too small).
- The blue line (intercept = 0, slope < 1) corresponds to predicted probabilities that are too extreme (that is, over-estimating risk for high-risk patients and under-estimating it for low-risk patients).
- The purple line (intercept = 0, slope > 1) corresponds to predicted probabilities that are too moderate (that is, under-estimating risk for high-risk patients and over-estimating risk for low-risk patients).

Supplementary Figure 2. Summary of predicted probabilities by model

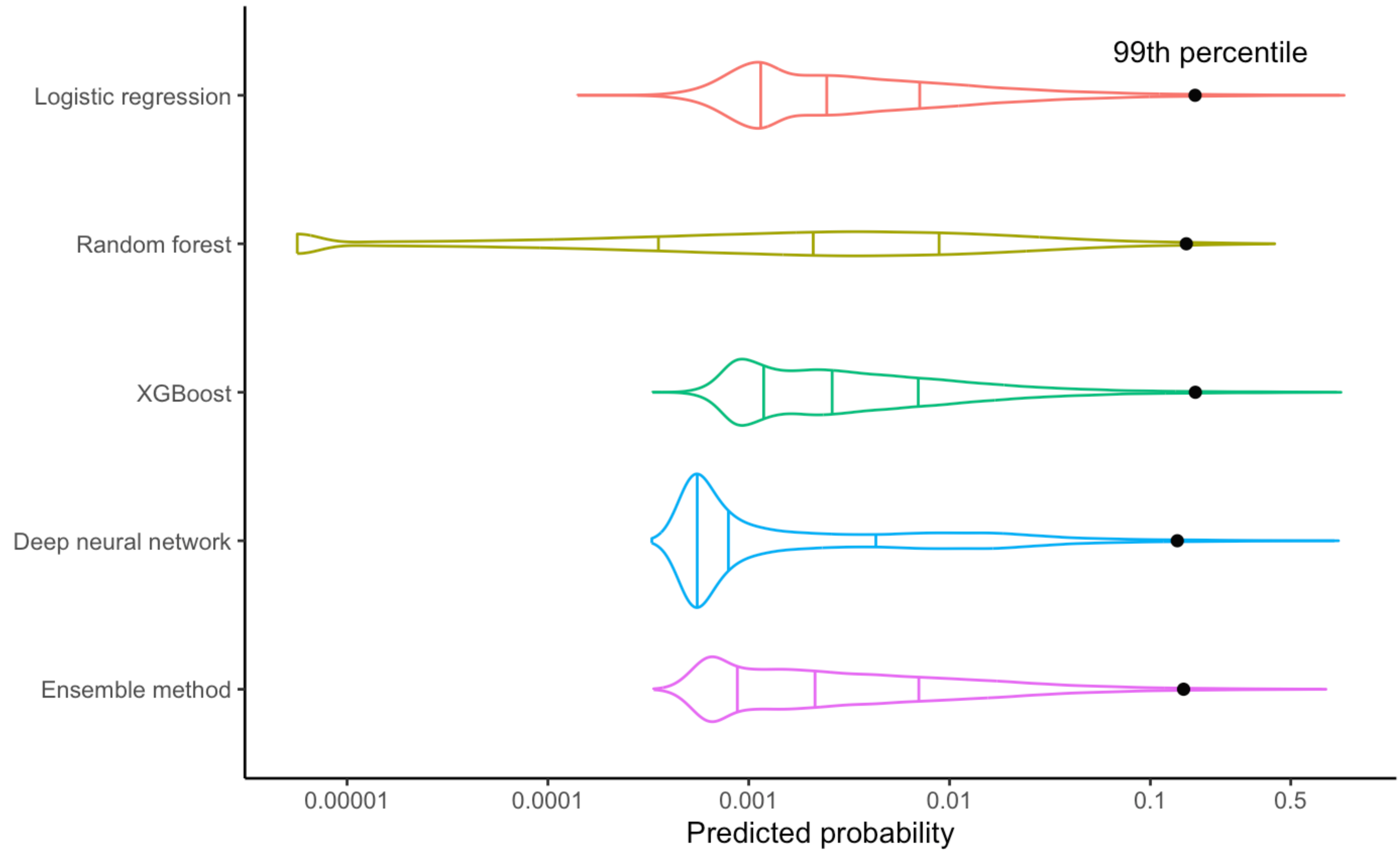

Supplementary Figure 3. Correlation in predicted probabilities between models

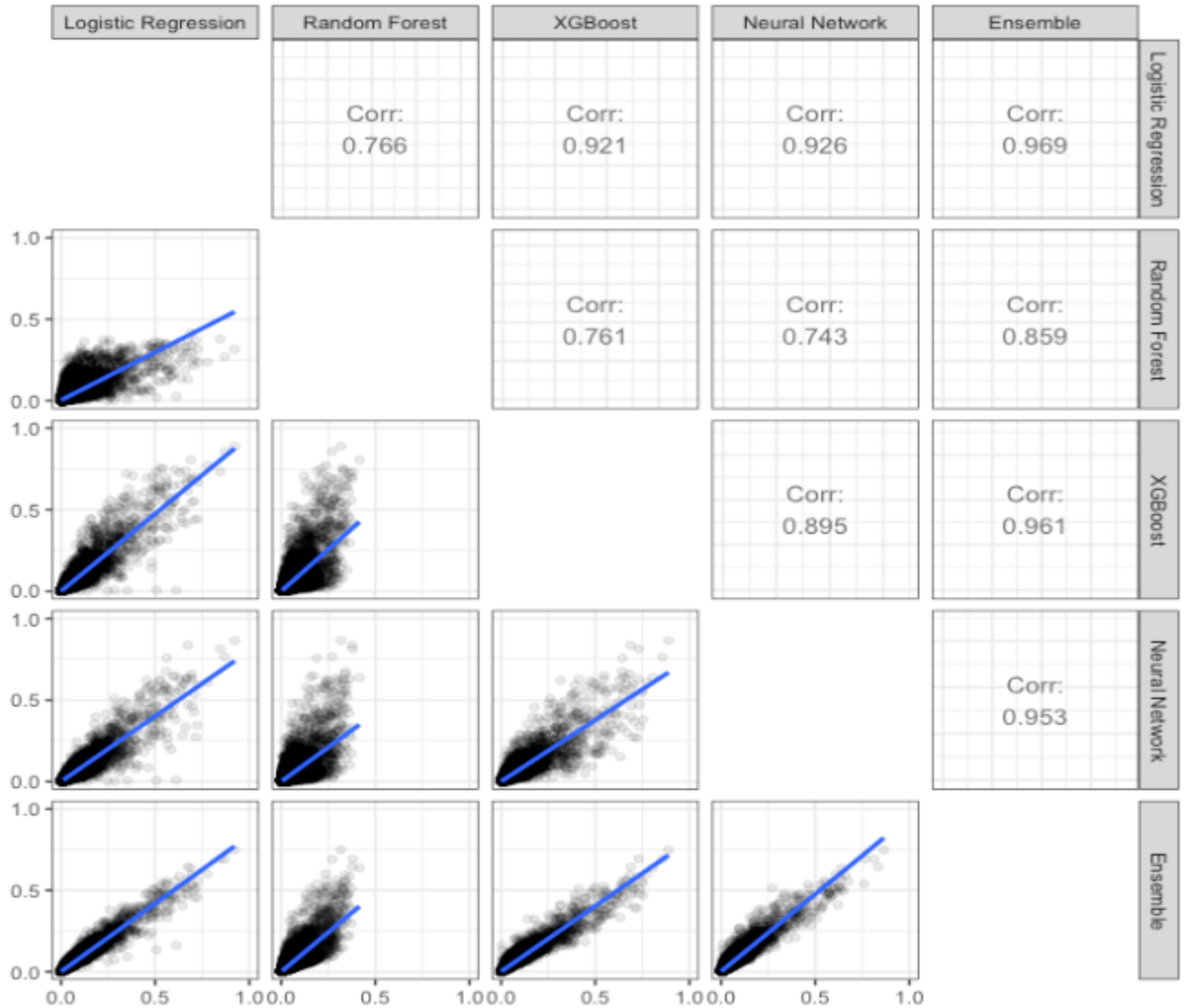

Overall, the predicted probabilities from all models were highly correlated, with correlation values exceeding 0.74 (Supplementary Figure 3). However, the predicted probabilities obtained via random forest were not as strongly correlated with those obtained via other models, except for the ensemble model. On the other hand, the predicted probabilities obtained via logistic regression, XG boost, and ensemble method had higher correlation (0.92-0.97), likely due to their similar distributions of predicted probabilities (Supplementary Figure 2).

Supplementary Figure 4. Non-parametric calibration curves on the (a) probability scale and (b) log-odds scale

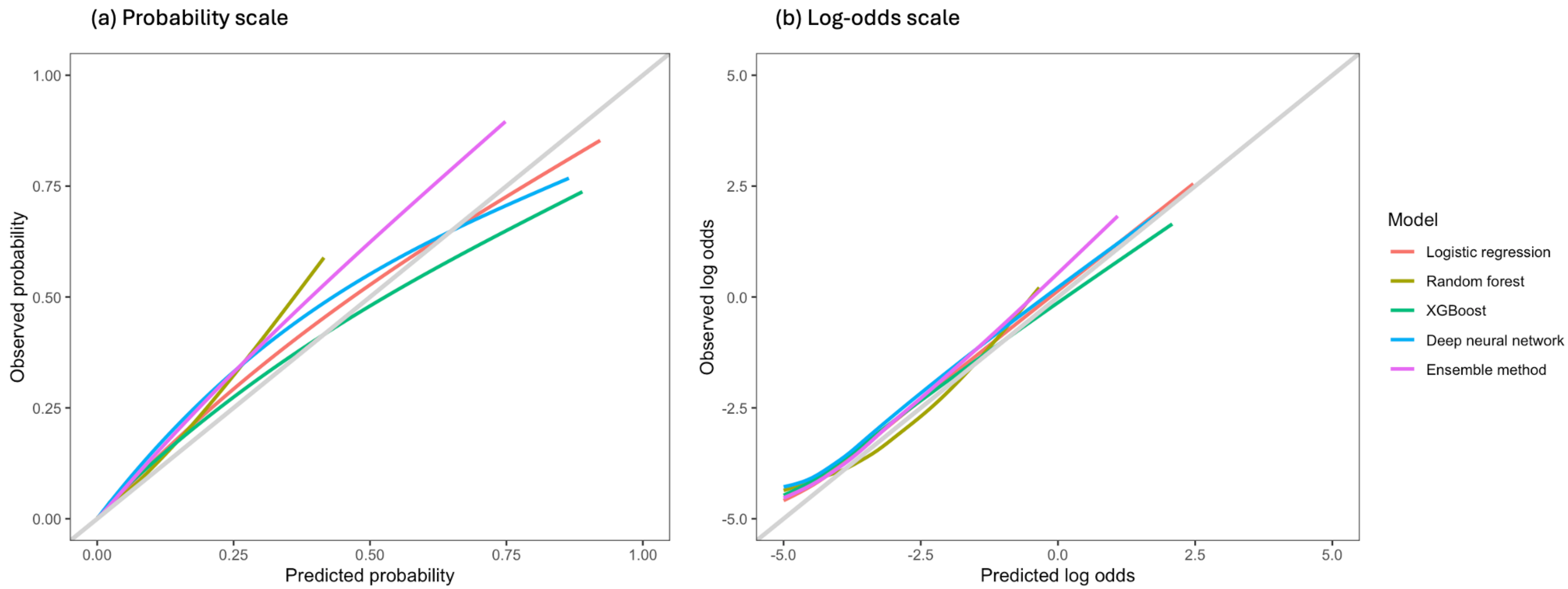

Unlike the parametric calibration curves in Figure 1, the non-parametric curves are shown only over the range of observed predicted risks. The gray 45-degree line corresponds to a calibration intercept of 0 and a calibration slope of 1 (i.e., perfect calibration).

Supplementary Figure 5. *C* statistics among key subgroups

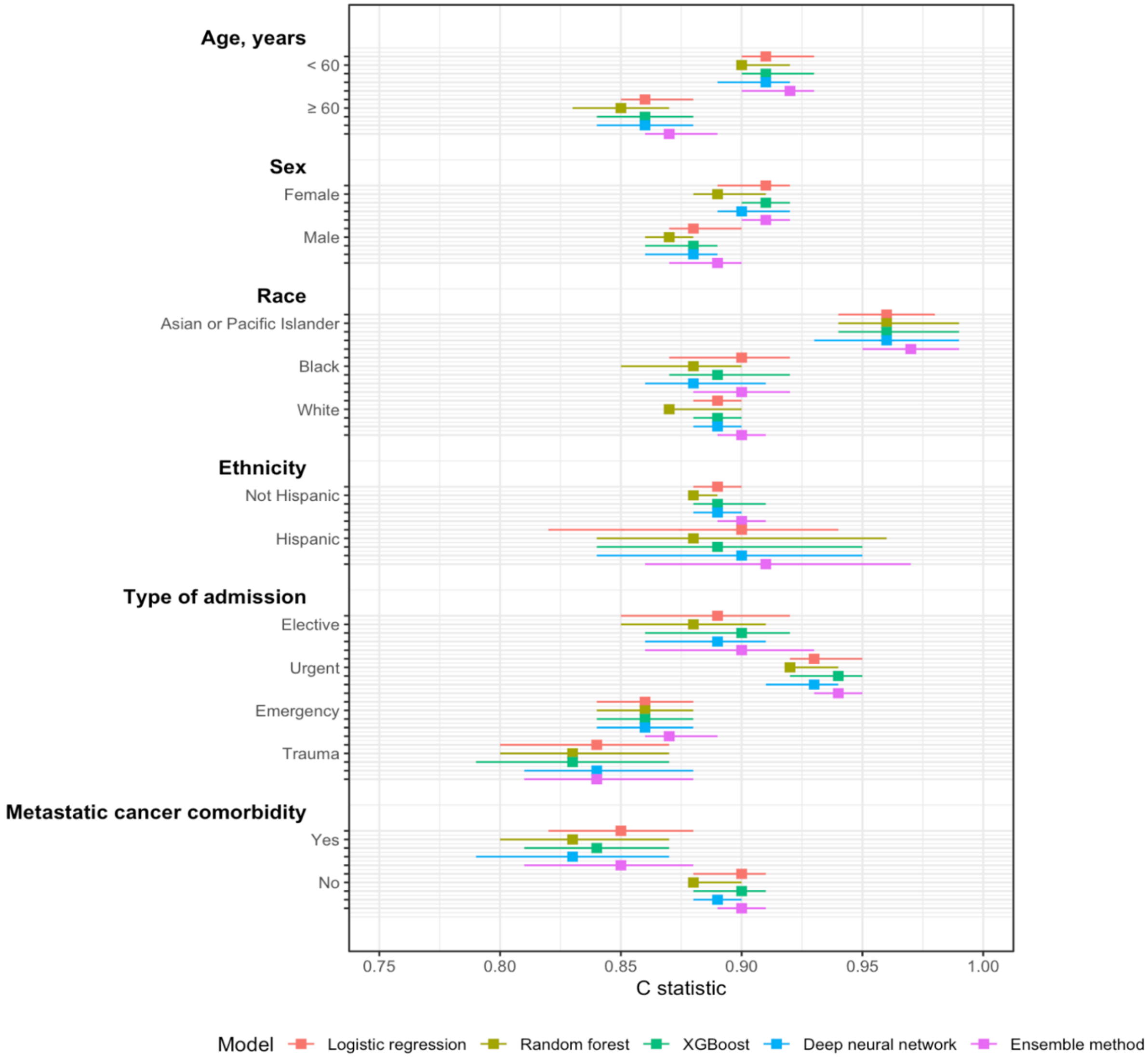

Supplementary Figure 6. Calibration slopes among key subgroups

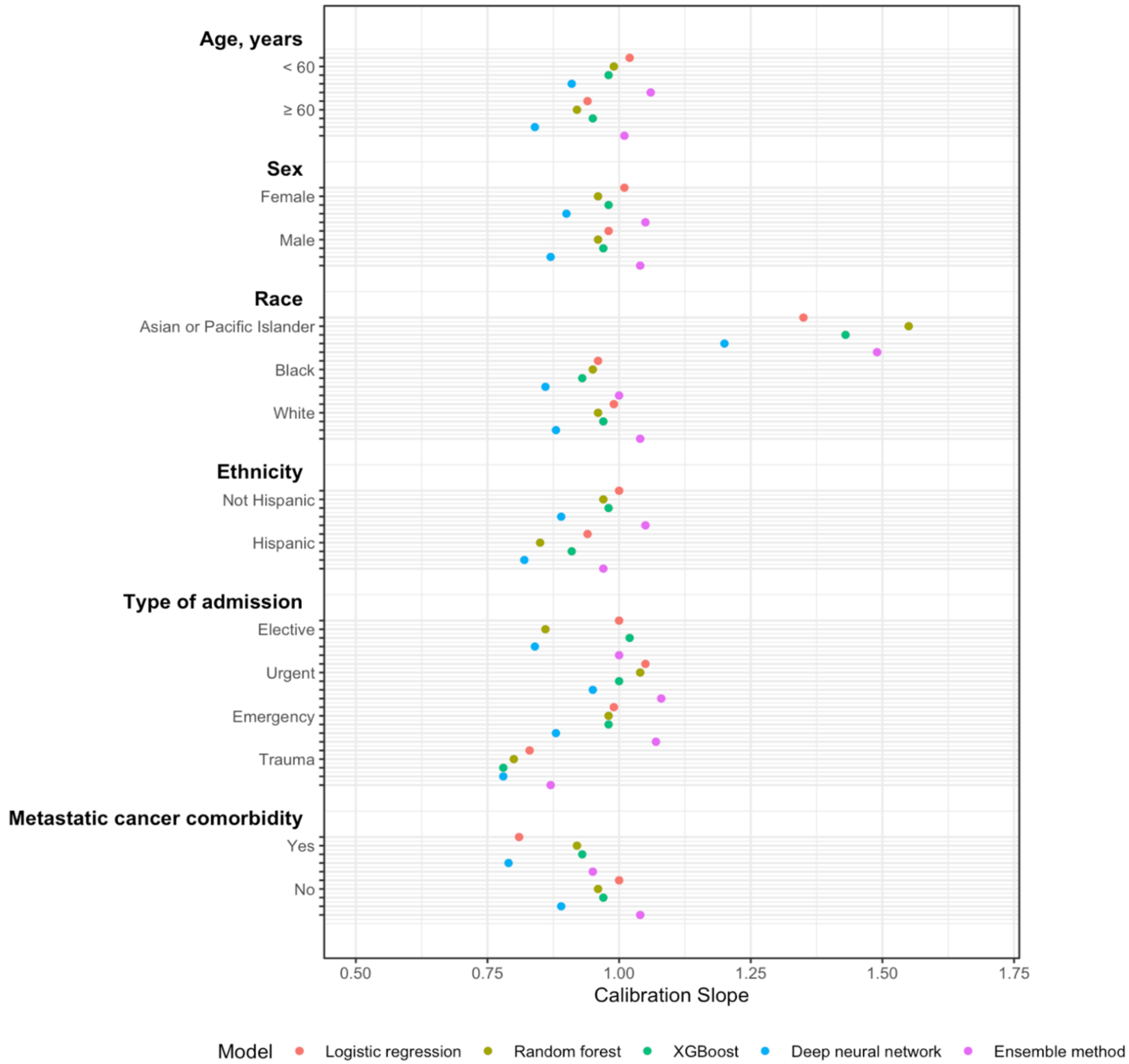

Supplement: Supplementary file 1 [file JHM-21-1218-s001.pdf]
